# Straightplasty, a Limb Salvage Procedure in Malignant and Aggressive Bone Tumors of Lower Extremities: A Retrospective Analysis

**DOI:** 10.7759/cureus.15294

**Published:** 2021-05-28

**Authors:** Pankaj Kumar Sharma, Zile Singh Kundu, Umesh Yadav

**Affiliations:** 1 Department of Orthopaedics, All India Institute of Medical Sciences (AIIMS), Bathinda, IND; 2 Department of Orthopaedics and Rehabilitation, Post Graduate Institute of Medical Sciences, Rohtak, IND; 3 Department of Orthopaedic Surgery, Positron Multispeciality Hospital, Rohtak, IND; 4 Department of Orthopaedic Surgery, Post Graduate Institute of Medical Sciences, Rohtak, IND

**Keywords:** straightplasty, malignant bone tumours, limb salvage procedures, functional evaluation, psychosocial factors, rotationplasty, resection arthrodesis

## Abstract

Introduction

The management of malignant bone tumors of lower extremities involves various modalities, which depend not only on local and systemic affection but are also affected by psychosocial factors. The purpose of this study was to evaluate functional and psychosocial outcomes in patients with non-salvageable lower limbs having malignant or aggressive benign bone tumors of distal thighs, who were treated with a technique called straightplasty.

Material and methods

We enrolled 20 patients of non-salvageable primary malignant or aggressive benign bone tumors around the knee. Out of these, 15 patients were followed and evaluated in view of functional and clinical outcomes having a minimum of 22 months of final follow-up.

Results

A total of 15 patients (8 males, 7 females) having a non-salvageable lower limb with a mean age of 20.53 years (range, 12 to 45 years), who were managed with straightplasty and followed for a mean duration of 31.73 months (range 22 to 72 months) were evaluated clinico-radiologically, and the functional outcomes were measured by Enneking’ s method. The surgical procedure is simple and better in terms of functional outcomes than other procedures described in the literature, while it is observed as psychosocially more acceptable in developing nations, especially in the Indian context. Most of the parameters are comparable to rotationplasty and above-knee amputation, whereas it is less technically demanding and satisfying due to the straight limb rather than the rotated leg in rotationplasty.

Conclusion

We recommend straightplasty as an alternative to rotationplasty or above-knee amputation in patients having malignant or aggressive benign tumors around the knee joint and where limb salvage procedures are not feasible.

## Introduction

Sarcomas as a malignant entity account for approximately 1 % of all adult malignancies [1]. Overall, soft tissue sarcomas are found in middle-aged and older patients, whereas malignant bone tumors predominantly occur in the younger age groups (adolescent and young age) [2-3]. With the dynamic evolution of chemotherapy and radiotherapy along with the invention of advanced treatment options, these management regimens have profoundly changed the recent scenario of progression of the disease, thus remarkably improving the morbidity and survival rates of patients [4-6]. There are various surgical modalities for bone malignancy, including limb salvage reconstruction surgeries, resection reimplantation either irradiated (ECRT; extracorporeal radiotherapy) or not, endoprosthetic devices, rotationplasty, arthrodesis of joint, and amputation of the diseased limb [3]. Limb salvage outcomes are increased in up to 80%-95% of cases with these neoadjuvant regimens along with surgical procedures [4,7]. The rotationplasty technique considered an alternative to amputation as a partial limb salvage procedure was explained and popularised by Van Nes for congenital proximal femoral deficiency (PFFD) but the very first documentation in literature was done for an ankylosed tubercular knee joint in 1930 [3,8]. It is a well-established, time-tested procedure for malignant bone tumors with a good long-term follow-up in view of functional outcome and survival of the patient. We describe here an innovative technique called straightplasty (modified resection arthrodesis) for the treatment of malignant or aggressive bone tumors around the knee. The current study elaborates the surgical technique and long-term functional and psychosocial outcomes with this procedure and discusses the advantages of this procedure over other methods of treatment.

## Materials and methods

We enrolled 20 patients managed in the past 11 years (from January 2006 to December 2016), who underwent straightplasty procedures at the orthopedics oncology unit of a tertiary care center and followed up for a minimum of 22 months. Subjects with malignant or aggressive benign bone pathology of the knee, who could not afford the cost of an endoprosthesis, not giving consent for above-knee (A/K) amputation, and who could accept a straight though shortened limb, were included in the present study. Patients who presented with distance metastasis, non-ambulatory status, a non-functional or non-sensate limb with the neurovascular invasion of a tumor, severe cachexia, and not given consent for procedures were excluded from the study. All the patients presented with gross swelling and pain around the knee joint or distal thigh with different morbidities, including painful limp and difficulty in walking in 18 patients (90%). Three patients presented with pathological fractures of the lower end of the femur with trivial trauma. All the patients were investigated with radiographs of limbs and chest, ultrasonography of abdomen and pelvis, magnetic resonance imaging (MRI) of limb, bone scan and computed angiography for evaluation of pathology, and metastatic workup of malignancy. All the patients who had no evidence of distance metastasis underwent bone biopsy, which confirmed the diagnosis by histopathology examination (HPE). Histopathologically proven patients were enrolled for preoperative neoadjuvant chemotherapy and surgical intervention was carried out at around four weeks after the three doses of the primary chemotherapy regimen. Patients were counseled about all the available surgical modalities in the institute and informed written consent was taken for all surgical procedures and publication. The patient’s preoperative demographic characteristics and disease progression after surgical interventions have been summarized in Table 1.

**Table 1:** Patients demographic characteristics, disease progression, and final outcomes A/K: above knee; GCT: giant cell tumor

S.N.	Age/ sex	Diagnosis	Duration (months)	Metastasis	Chemotherapy (pre/post)	Post-operative progression of the disease	Final follow-up (months)
1	14/M	Osteosarcoma	3	No	Yes/Yes	Superficial infection antibiotics, dressing	27
2	16/M	Osteosarcoma	2.5	No	Yes/Yes		28
3	36/F	Aggressive GCT	5	No	No	Pathological fracture femur, wound necrosis	48
4	12/F	Ewing’s sarcoma	3.5	No	Chemo/Radio		72
5	15/M	Osteosarcoma	2	No	Yes/Yes		36
6	21/M	Osteosarcoma	2.5	Lung	Yes/Yes	H/o chronic smoker, multi-organ failure	Died at 12 months (cardio-respiratory arrest)
7	18/F	Ewing’s sarcoma	3	No	Chemo/Radio	Sup. Infection managed by antibiotics, dressing	30
8	13/F	Osteosarcoma	4	No	Yes/Yes		28
9	45/M	Malignant fibrous histiocytoma (MPH)	9	No	No	Osteomyelitis managed by debridement and intravenous antibiotics	22
10	22/M	Osteosarcoma	4	No	Yes/Yes	Systemic toxicity of chemotherapy	Cardiac failure (3 m)
11	16/F	Osteosarcoma	3.5	No	Yes/Yes		26
12	13/M	Osteosarcoma	3	No	Yes/Yes	Recurrence managed by A/K amputation	24
13	17/F	Osteosarcoma	4	No	Yes/Yes		22
14	15/F	Ewing’s sarcoma	3	No	Chemo/Radio		30
15	14/M	Osteosarcoma	2.5	Lung	Yes/Yes	Cardio-respiratory dysfunction	Died at six months
16	12/M	Osteosarcoma	3.5	No	Yes /Yes		25
17	15/M	Osteosarcoma	3	No	Yes/Yes		27
18	14/F	Osteosarcoma	2.5	No	Yes/Yes	Recurrence managed by A/K amputation	15
19	22/M	Chondrosarcoma	8	Local recurrence	No	Sup. infection	26
20	42/M	Chondrosarcoma	7.5	No	No	Wound necrosis, sup. infection	29

Functional outcomes were measured by Enneking's functional evaluation system, which consists of clinical tests performed by surgeons and a standard questionnaire for patients [9]. It incorporates six categories of variables with numerical values of 0 to 5 for each. For the lower extremities, the measured variables are pain, emotional acceptance, functions, support, walking, and gait components. Biostatistical analysis of data compiled with Prism Five software (GraphPad, San Diego, CA) presented as categorical (percentage) and normally distributed variables (mean and standard deviation). Quantitative variables were compared with the paired student's t-test and considered significant if the p-value is less than .05 (p<.05).

Operative procedure

**Figure 1:**
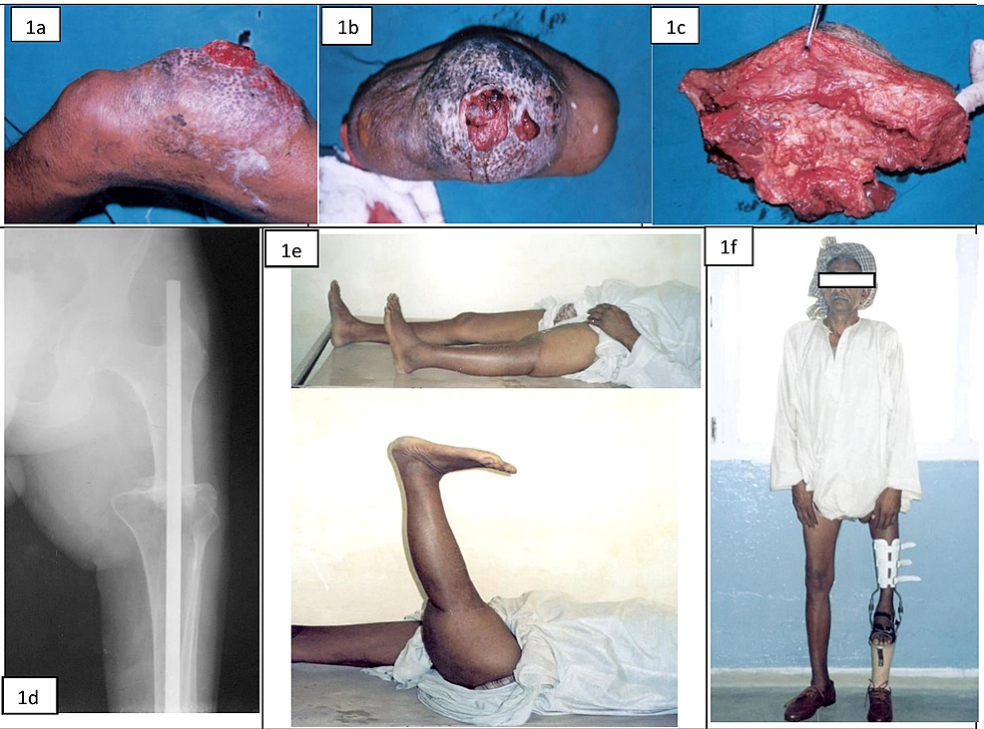
A 45-year-old male with a diagnosis of malignant fibrous histiocytoma (MPH) distal thigh (First row) 1a (side view) and 1b (front view), showing clinical pictures of angiomatoid type variant; 1c, excised tumoral mass with soft tissue components; (second row); 1d, radiographs showing well-healed osteosynthesis of the lower limb with intramedullary nailing; 1e, clinical picture with full functional shortened straight limb; 1f, showing the patient standing with the custom-made extension prosthesis.

All the patients were operated on under regional/epidural anesthesia, either in supine or semi-lateral position, with informed and written consent. Surgical exposure was done as described in the literature for rotationplasty and resection arthrodesis with some modifications [10-11]. The surgery included wide resection with adequate margins after applying the pneumatic tourniquet in the supratrochanteric area for the lower femoral tumors. The high tourniquet allowed less blood loss, a clear surgical field thus less surgical time, and easy handling or isolation of neurovascular bundle from the tumor mass. Meticulous soft tissue handling and flap raise were done for adequate closure after surgery. Surgical steps were simpler than rotationplasty, as there was no need for rerouting of all the muscles and neurovascular bundles of thigh and leg. There was no need for tibial rotation, so adequate alignment of the bone ends of the femur and tibia made arthrodesis easier using either dynamic compression plate (DCP)/locking plate or intramedullary nailing (IMN). IMN implants are more stable and safer for fixation and so preferred over plating for arthrodesis. A trough was made in the broad tibial metaphyseal end, which allowed more contact area for a union at the arthrodesis site. All the patients were immobilized in a cylindrical slab from the groin to the lower end of the leg while the ankle joint and foot were kept free for early mobilization. Intravenous antibiotics were given for the initial five days followed by oral broad-spectrum antibiotics up to suture removal (12 to 14 days). After adequate healing of the surgical wound (3 to 4 weeks), postoperative chemotherapy was started as advised by the tumor board of the institute. A customized extension prosthesis made by composite material (PVC) in the prosthetics department of the institute was provided to patients after six to eight weeks of surgery. A patient aged 45 years, having malignant fibrous histiocytoma of the distal thigh (angiomatoid type), managed with the straightplasty procedure and postop rehabilitation with a customized prosthesis, has been shown in Figure 1.

Interdisciplinary physiotherapy was started as soon as possible with patient compliance, including ankle/foot mobilization and bedside movements. Special balance and gait training were provided by the physiotherapist once the prosthesis was implanted over the limb [12].

## Results

**Figure 2:**
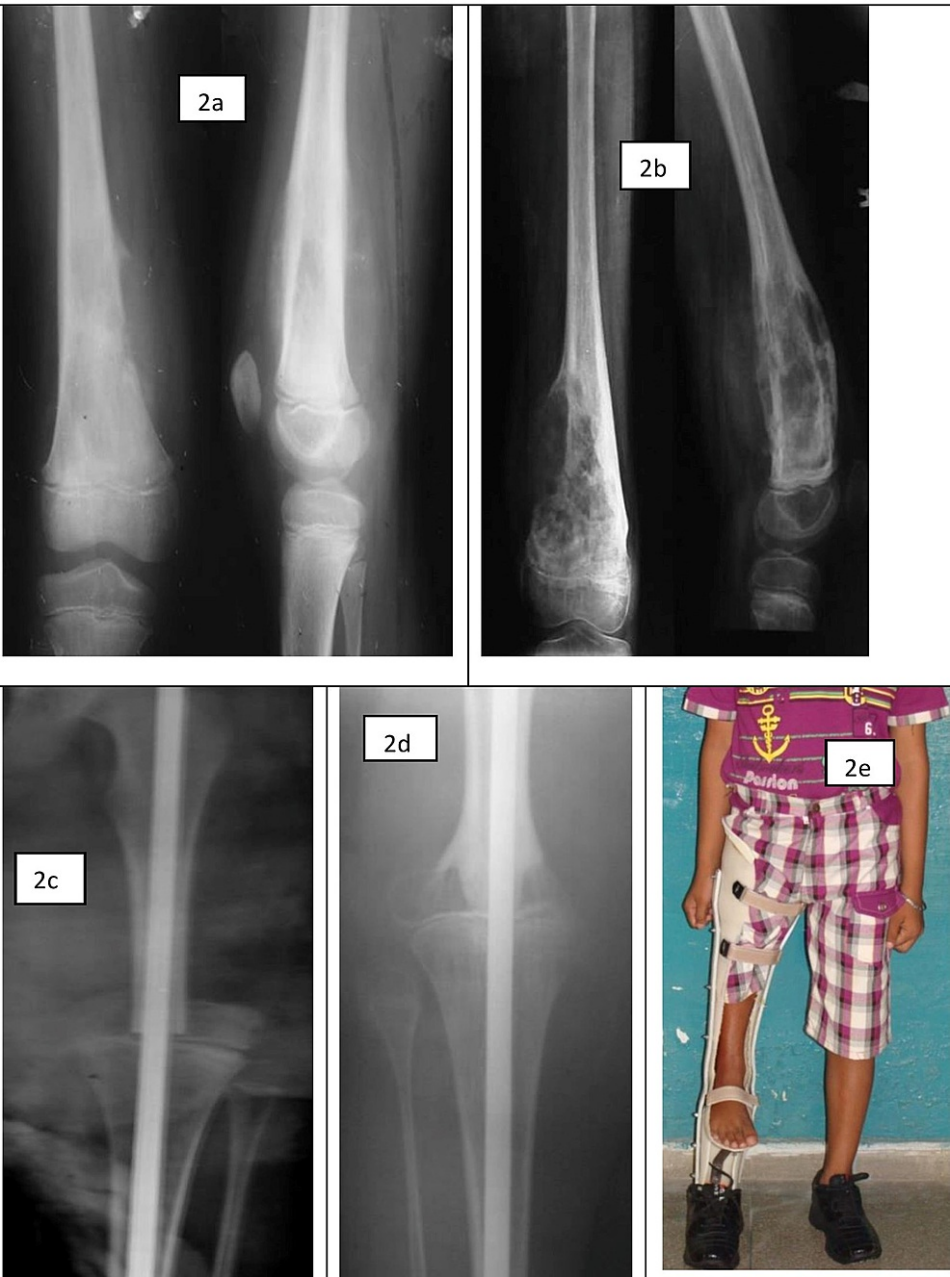
A 12-year-old boy with a diagnosis of osteosarcoma of the distal thigh (First row) showing radiographs (anteroposterior and lateral views) of the femur with an osteolytic lesion, pre-chemotherapy (2a) and post-chemotherapy films (2b), (second row) radiographs showing postoperative osteosynthesis of the partially salvaged limb with intramedullary nailing, immediate postop with adequate alignment (2c); and one-year follow-up with callus formation/healed bone ends (2d), clinical picture with the custom-made extension prosthesis in functional position (2e).

We enrolled a total of 20 patients (12 males, 8 females) but did not include five patients (2 recurrences and 3 deaths) for final evaluation of the cohort so only 15 patients were free of any sign or symptom of the disease for the mean duration of postoperative follow-up of 31.73 months (range 22 to 72 months) were included. The male: female ratio was 8:7 with a mean age of 20.53 years (range, 12 to 45 years) at first presentation. All the patients were presented primarily or referred from peripheral centers with a mean duration of symptoms of 4.3 months (range 2 to 9 months) at first presentation. Osteosarcoma was the most common pathology in 13 patients followed by Ewing sarcoma in three, chondrosarcoma in two, aggressive giant cell tumor (GCT), and malignant fibrous histiosarcoma (MPH) in one patient each. The mean operative time was 174.33 minutes (range 135 to 240 minutes) with an average blood loss of 600 ml (range 500 to 800 ml). They were ambulated with a customized extension prosthesis assembled in the institute, without any external walking aid. A young patient having Ewing sarcoma of the distal thigh managed with straightplasty and rehabilitated adequately with prosthesis has been shown in Figure 2.

Four patients developed superficial infections (SI), which were resolved with aseptic dressings and antibiotic coverage within one week. One patient developed osteomyelitis and managed with debridement and intravenous antibiotics for two weeks. Two patients developed local recurrences within two years and managed by above-knee amputation, so not included for final functional evaluation of the limb. Two patients having osteosarcoma of limbs died of lung metastases at six months and 12 months, respectively. One patient died of cardiac failure at three months postoperative, which might be due to systemic toxicity of chemotherapy. All the patients experienced mild pain on prolonged standing and brisk walking, which was relieved by rest and medication. There was no single event of implant failure at the final follow-up. Clinically, as well as radiologically, there were no signs of local recurrence in 15 patients. Functional outcomes were measured by Enneking's method and the estimated mean score was 56.9% (range 46.7 to 60%). Postoperative functional evaluation with various parameters of Enneking's method at final follow-up has been documented in Table 2.

**Table 2:** Functional outcomes of limbs after surgical procedures estimated by Enneking’s method A/K: above knee

Patient profile	Supports	Walking	Gait	Pain	Function	Emotional acceptance	Total score (%), outcome
1	3	3	1	4	4	3	18/30 (60)
2	3	3	1	5	3	3	18/30 (60)
3	3	3	1	2	3	4	16/30 (53.3)
4	3	3	1	4	3	3	17/30 (56.7)
5	3	3	1	5	3	3	18/30 (60)
6	NA	NA	NA	NA	NA	NA	Died at 12months
7	3	3	1	1	3	3	14/30 (46.7)
8	3	3	1	2	4	4	17/30 (56.7)
9	3	3	1	4	3	3	17/30 (56.7)
10	NA	NA	NA	NA	NA	NA	Died at 3 months
11	3	3	1	4	3	3	17/30 (56.7)
12	NA	NA	NA	NA	NA	NA	A/K amputation
13	3	3	1	3	3	3	17/30 (56.7)
14	3	3	1	4	3	3	17/30 (56.7)
15	NA	NA	NA	NA	NA	NA	Died at 6 months
16	3	3	1	5	3	3	18/30 (60)
17	3	3	1	5	3	3	18/30(60)
18	3	3	1	1	3	3	A/K amputation
19	3	3	1	4	3	3	17/30 (56.7)
20	3	3	1	2	4	4	17/30 (56.7)

## Discussion

The knee joint is one of the most common joints affected by bone tumors. Malignant and aggressive tumors need special attention for long-term outcomes, so decision-making is of utmost importance for the selection of the procedure. Extensive local lesions with secondary changes, widespread metastases, non-functional limbs, non-sensate limbs, neurovascular bundle invasion, and severe cachexia are conditions for patient demand for amputation rather than limb salvage surgeries. These tumors need wide excision of malignant mass along with the sacrifice of adjacent soft tissues and osseous elements, thus creating large bone defects, resulting in severe limb length discrepancy. Major challenges for these pathologies are resection, reconstruction, complication, and their management in an adequate manner. There are successful functional and cosmetic outcomes with limb salvage surgeries, including massive composite allografts, vascularized fibular grafts, and, recently, reconstruction using modular endoprostheses [7,13-15]. These results are satisfying in various aspects but are associated with a number of complications; infection is the most common and disastrous and may need revision surgeries in many cases [14]. Endoprostheses are highly associated with infections and reinfections of primary and revision surgeries respectively, resulting in high morbidities and needing limb-sacrificing surgeries in the form of amputations or even hip disarticulation in extreme infections [14-16]. A young patient, with a failed, infected endoprosthesis who was treated for aggressive giant cell tumor (GCT) of the distal thigh, previously managed efficiently with straightplasty, has been documented in a case report [16].

Rotationplasty was explained initially for malignant bone tumors by Salzer et al., which was further elaborated by Winkelmann et al. for widespread locations of the lower limb [17-19]. As described earlier, rotationplasty includes a 180-degree rotation of the leg over the thigh, resulting in the conversion of the ankle to functioning as a knee joint. Straightplasty follows the same mechanism of function, except for rotation of the limb, resulting in a short straight limb. The new knee made by the straight ankle-like rotated ankle in rotationplasty accommodates the patient’s knee mechanism during movement and control coordination of limbs during walking [16,20-21]. As it is straight rather than rotated ankle in rotationplasty, it might be very easy to adapt the modified limb position and anatomy, changed with the straightplasty procedure. The sensate foot is better in view of tolerance and accommodating the prosthesis than an amputated knee or disarticulated hip, so it can move faster than them [21-22]. One more advantage with that is that the required prosthesis is shorter than the amputated limb. It may consume less energy to accelerate muscles of extremity, thus walking distance and speed may be better than other procedures. A straight limb is more psychologically sound than an amputated or inverted limb for patients, especially in the context of developing countries, where social stigmas and superstitious values are abundant regarding an inverted or absent limb [16]. One disadvantage of the procedure is that patients lost the benefits of using the ankle as a newer knee joint, which is described for rotationplasty. The patient may keep intact their proprioception of the joint along with the psychological satisfaction of a salvaged foot, which motivates the patient to accommodate with prosthetic fitting [16]. As mentioned in Enneking’s functional outcome evaluation, emotional acceptance is affected by patient profile and socioeconomic status, hence subjects were found more satisfied (non-significant, p > 0.05) emotionally with a straight limb than a rotated one [9].

The same procedure was performed by the author for the failed endoprosthesis to salvage a partial functional limb [16]. There may be the possibility of achieving limb length by a surgical procedure including distraction osteogenesis in the future, which seems more feasible in straight limbs rather than in rotated [23]. Intraoperatively there are less technically demanding steps to address the rotation of the tibia and soft tissue alignment in a non-anatomical manner for straightplasty than rotationplasty. Additionally, it also reduces surgical time, the possibility of blood loss, and other complications related to the manipulation of soft tissue and neurovascular structures. Moreover, complications with amputated stumps, including skin necrosis, bone growth at ends/spur formation, phantom sensations, and neuroma formation, are not found. In developing countries, especially in the Indian context, patients have a different type of socio-economic status and work expectations according to their daily activities of living so treatment should be cost-effective, durable, and innovative for different age groups. In addition, there is the social stigma of the appearance of a ghost (*bhoot*) from Indian folklore, whose feet are facing backward, which is also associated with rotationplasty patients, so a straight functional but short limb is more acceptable socially as in straightplasty.

Limitations and strength

There is a fewer number of similar and comparative studies in the literature to evaluate functional outcomes. The sample size of the study is small and it is conducted retrospectively so further prospective studies with a large sample size may be suggested for better functional evaluation in the long term. This study is one of the first to incorporate this procedure to manage malignant tumors so it may be considered a pioneer for future studies. All the patients were treated by a single senior orthopedic oncology surgeon while rehabilitated by a custom-made prosthesis in the same institute, so there are fewer chances of errors and dropouts in treatment and evaluating various parameters at different follow-ups.

## Conclusions

We concluded that straightplasty may be used as an alternative to rotationplasty or above-knee amputation in patients, where a complete limb-salvaging procedure is not possible. Additionally, there may be more emotional acceptance with this procedure than with others due to the straight but intact short limb. The procedure also reflects comparable or better functional outcomes than above-knee amputation, which is especially concerned with the psychosocial aspect. We need a large cohort of patients or a multicenter trial in future studies to validate this method.
